# A Quantitative Diffuse Reflectance Imaging (QDRI) System for Comprehensive Surveillance of the Morphological Landscape in Breast Tumor Margins

**DOI:** 10.1371/journal.pone.0127525

**Published:** 2015-06-15

**Authors:** Brandon S. Nichols, Christine E. Schindler, Jonathon Q. Brown, Lee G. Wilke, Christine S. Mulvey, Marlee S. Krieger, Jennifer Gallagher, Joseph Geradts, Rachel A. Greenup, Jesko A. Von Windheim, Nirmala Ramanujam

**Affiliations:** 1 Department of Biomedical Engineering, Duke University, Durham, NC, United States of America; 2 Department of Pathology, Duke University Medical Center, Durham, NC, United States of America; 3 Department of Surgery, Duke University Medical Center, Durham, NC, United States of America; 4 Department of Surgery, The University of Wisconsin School of Medicine and Public Health, Madison, Wisconsin, United States of America; 5 Zenalux Biomedical, Research Triangle Park, NC, United States of America; 6 Department of Biomedical Engineering, Tulane University, New Orleans, LA, United States of America; 7 The Division of Environmental Sciences and Policy, Duke University, Durham, NC, United States of America; Institute of Physics, University of Zurich, SWITZERLAND

## Abstract

In an ongoing effort to address the clear clinical unmet needs surrounding breast conserving surgery (BCS), our group has developed a next-generation multiplexed optical-fiber-based tool to assess breast tumor margin status during initial surgeries. Specifically detailed in this work is the performance and clinical validation of a research-grade intra-operative tool for margin assessment based on diffuse optical spectroscopy. Previous work published by our group has illustrated the proof-of-concept generations of this device; here we incorporate a highly optimized quantitative diffuse reflectance imaging (QDRI) system utilizing a wide-field (imaging area = 17cm^2^) 49-channel multiplexed fiber optic probe, a custom raster-scanning imaging platform, a custom dual-channel white LED source, and an astronomy grade imaging CCD and spectrograph. The system signal to noise ratio (SNR) was found to be greater than 40dB for all channels. Optical property estimation error was found to be less than 10%, on average, over a wide range of absorption (μ_a_ = 0–8.9cm^-1^) and scattering (μ_s_’ = 7.0–9.7cm^-1^) coefficients. Very low inter-channel and CCD crosstalk was observed (2% max) when used on turbid media (including breast tissue). A raster-scanning mechanism was developed to achieve sub-pixel resolution and was found to be optimally performed at an upsample factor of 8, affording 0.75mm spatially resolved diffuse reflectance images (λ = 450–600nm) of an entire margin (area = 17cm^2^) in 13.8 minutes (1.23cm^2^/min). Moreover, controlled pressure application at the probe-tissue interface afforded by the imaging platform reduces repeated scan variability, providing <1% variation across repeated scans of clinical specimens. We demonstrate the clinical utility of this device through a pilot 20-patient study of high-resolution optical parameter maps of the ratio of the β-carotene concentration to the reduced scattering coefficient. An empirical cumulative distribution function (eCDF) analysis is used to reduce optical property maps to quantitative distributions representing the morphological landscape of breast tumor margins. The optimizations presented in this work provide an avenue to rapidly survey large tissue areas on intra-operative time scales with improved sensitivity to regions of focal disease that may otherwise be overlooked.

## Introduction

For most women with stage I or II breast cancer, a surgical procedure known as breast conserving surgery (BCS) is available as an alternative to a total mastectomy. Also known as a partial mastectomy or “lumpectomy,” BCS has been shown to be as effective as a mastectomy when combined with radiation therapy. BCS is therefore the more frequent surgical choice for patients with early stage breast cancer [1, 2]. The two primary objectives of BCS are to preserve as much of the normal breast as possible while simultaneously removing the entire tumor. Historically, the standard of care at Duke University Medical Center (DUMC) requisitioned surgeons to remove a 2mm wide “margin” of disease-free tissue surrounding the malignant site. The post-operative margin status [negative = no disease in margin, close = disease present < 2mm from surface, positive = disease present at surface] has been shown to be predictive of the likelihood of local disease recurrence [3, 4]. Standard practice typically requires the margin status to be assessed post-operatively by a pathologist, reducing the likelihood of additional tissue resection during the initial surgery in the event of a positive or extremely close margin, possibly leaving residual disease within the patient and warranting a second surgery.

The clinical unmet need for an intra-operative tool to facilitate immediate margin assessment in BCS is significant: the American Cancer Society predicts 231,840 new cases of breast cancer among females in 2015 with an estimated 80% eligible for BCS and an estimated 38%-65% that will actually undergo BCS [2, 5]. Moreover, an estimated 25% of BCS recipients will be advised to have a second surgery due to the finding of malignant cells at or near the margin of the breast specimen at final pathology [6–10]. Medical centers in the United States seldom (<5%) leverage currently available intra-operative margin assessment techniques (cytologic or pathologic) due to the specialized resources required, such as a pathologist during surgery; the low availability and added cost of these resources to most outpatient clinics suggest a marginal reduction of the clinical burden and incidence of local recurrence. The current standard of practice leverages post-operative histopathology, wherein 4–5mm micrometer slices are taken from larger 3–4mm sections of the tumor margin and thus subject to practical sampling limitations. These sampling limitations are substantially more challenging intra-operatively where time is critical; strategic sampling informed by wide-field surveillance is necessary for the detection of small regions (<2mm) of residual disease amidst the relatively large (area = 10–100 cm^2^), mostly benign specimen background [11].

Quantitative diffuse reflectance imaging (QDRI) is an emerging modality that leverages diffuse reflectance spectroscopy (DRS) to accurately quantify the absorption and scattering properties of turbid media such as tissue. Scattering and absorption spectra (μ_a_(λ) and μ_s_’(λ), respectively) reveal morphological changes in tissue in the form of constituent absorber concentrations (such as hemoglobin, β-carotene, melanin, etc.) and scatterer size and density (such as nuclei, collagen, glands, etc.). DRS has recently been investigated for pre-cancer detection and cancer diagnostics [12–26], intraoperative tumor margin assessment [27–29], monitoring of tumor response to therapy [27, 30–32], and tissue oximetry [33]. An overview of recent progress in DRS for diagnosis, prognosis, and treatment of various cancers can be found in two review articles by Brown *et al*.[34], and Liu [35]. DRS has a penetration depth range from hundreds of micrometers to several millimeters in tissue; this range of perceptible information is ideal for breast tumor margin assessment as clear margins have been historically defined as no disease present at distances ≤ 2mm, although these criteria have been modified recently at DUMC and elsewhere [36]. DUMC now considers all margins not truly positive to be strictly negative; studies have shown that the long-term clinical outcome is no different for those who have re-excisions and those who do not when the margin status is “close” but not positive when followed up with radiation therapy [37]. The sensitivity to tissue morphology, penetration depth, and rapid acquisition speed afforded by modern technology suggest that an optically-based intra-operative margin assessment tool would be of great utility.

Our group has investigated several iterations of a multiplexed single point UV-VIS DRS probe that has been extensively validated in multiple phantom and clinical studies [18, 38–42]. The most recent embodiment consisted of an 8-channel (8ch) QDRI instrument arranged in a 4x2 array. The 8ch QDRI system was capable of imaging 1–3 margins of a resected lumpectomy breast specimen at a resolution of 5mm at a rate of 7–8 minutes per margin. The diffuse reflectance spectrum from each channel was analyzed with a feature extraction algorithm based on a fast, scalable Monte Carlo model developed by Palmer *et al*. (US 7,570,988) [18, 43, 44] and licensed to Zenalux, Inc., to quantitatively determine μ_a_(λ) and μ_s_’(λ). Brown *et al*. [45, 46] achieved a sensitivity and specificity of 74% and 86%, respectively, in detecting close or positive margins when the patient’s mammographic breast density (MBD) was considered *a priori*. The spectral information is used as a surrogate for tissue morphology, affording contrast between benign and malignant tissue and the respective subtypes. This strategy has provided a means to quickly survey 10s of cm^2^ tissue areas, quantitatively, on intra-operative time scales.

The differentiating feature of QDRI is derived from the ability to detect varying amounts of malignancy in the presence of benign tissue (which is highly variable across patients). One way to achieve this is by quantifying the margin “landscape” as a cumulative distribution function (CDF) of the ratio of β-carotene concentration to the magnitude of tissue reduced scattering ([β-carotene]/<μ_s_’>). The underlying premise formulates that malignancy perturbations will skew the derived distribution in a given direction relative to a completely benign distribution. We hypothesized, and have since verified with histopathological validation, that the [β-carotene]/<μ_s_’> reports on the relative amount of adipose to collagen, glands, and fibrous content, which is linked to the presence of residual disease [46–49].

The clinical utility of our spectral surveillance technique to detect shifts in histologic landscapes has motivated further improvement of this technology; we have developed a new 49-channel (7x7 array) QDRI system, hereafter referred to as the “C49 system”, with coverage area of 17cm^2^, affording complete single-margin surveillance (surface area typically 8–25cm^2^) in a spectral snapshot requiring 14 seconds or less, depending on the integration time necessary. The ability to simultaneously acquire data from 49 sites will allow us to optically assay considerably larger tissue areas than previously possible; in most cases, the entire specimen face can be imaged. Furthermore, we have developed a custom raster-scanning imaging platform with the following goals in mind: increasing the sensitivity to small focal positive regions on the margin through sub-millimeter sampling, eliminating confounding user-specific error stemming from the highly variable pressure when the probe is manually applied to the specimen, and reducing false positives resulting from compression of the margin thickness during measurements. Pressure control at the probe-tissue interface is realized through the use of 4 discrete load sensors subsequently converted to pressure based on the area of the probe and the contact area of the sensors. For the clinical data detailed in this work, 10mmHg of pressure was applied to each margin; an investigational study on animal tissue revealed 10mmHg to be optimal in the sense that contact was sure to be established without risk of compression of the natural, *ex vivo* margin thickness. Sub-pixel sampling is achieved by means of incremental raster-scanning of the probe, resulting in a series of spectral snapshots that are stitched into a hi-resolution QDRI map; this capability enables full margin surveillance at a resolution of 0.75mm at a rate of 1.23cm^2^/min and is reproducible to within 1%. Finally, we present a preliminary 20-patient high-resolution eCDF analysis to demonstrate the utility of this technology for breast tumor margin landscape characterization.

## Methods

### System Design

#### Ethics Statement

This study was performed in strict accordance with a protocol approved by the Duke University Institutional Review Board (Pro00007857). Patients over age 18 undergoing BCS granted written consent under the approved clinical protocol.

#### Instrumentation

**Fig 1:**
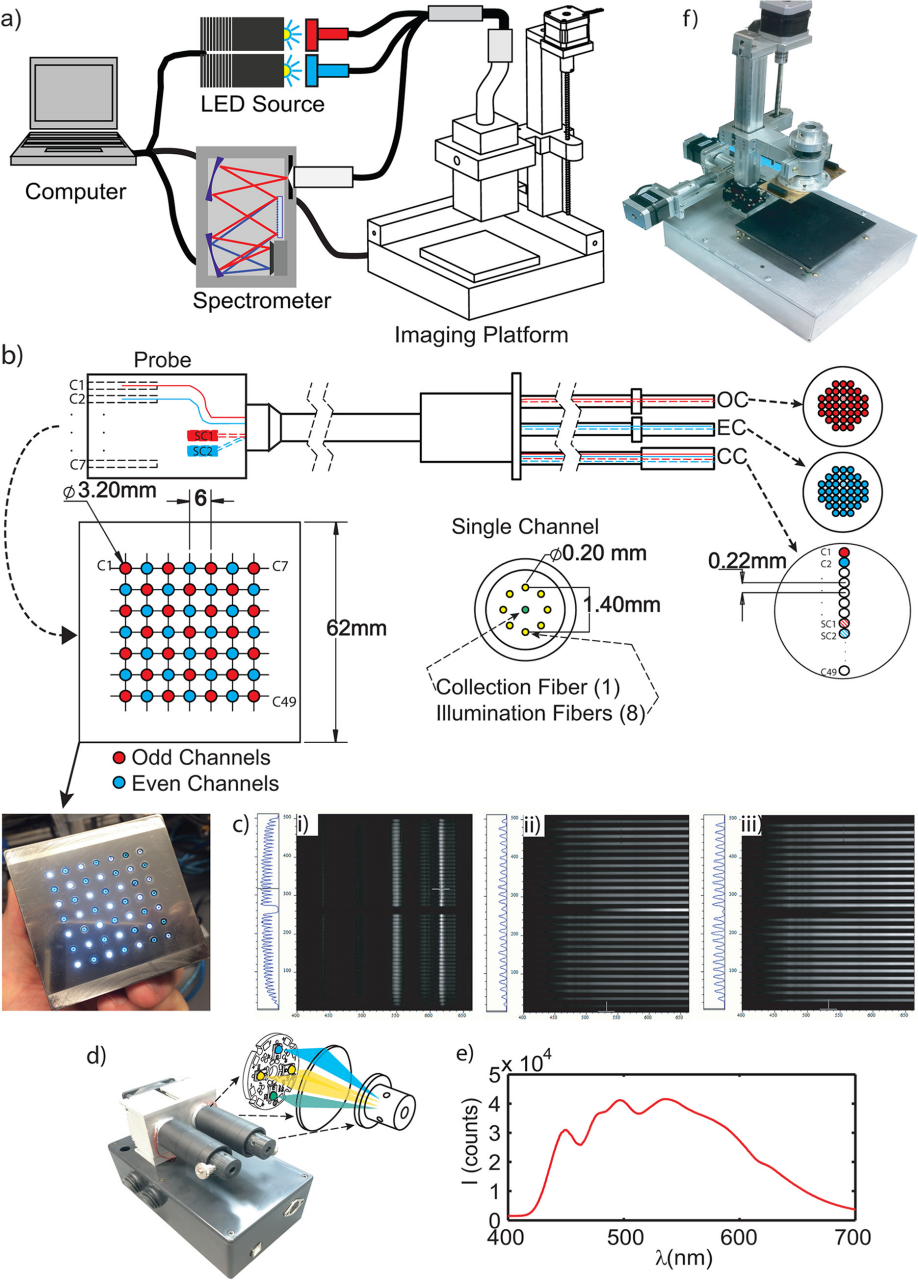
Imaging Probe and System Design. a) A diagram of the integrated system, b) Digital image and engineering sketch of the 49 channel QDRI probe design, consisting of a 7x7 grid of fiber optic channels each comprised of 8 illumination fibers and a single, central detection fiber (8 around 1). The aggregate collection of illumination fibers are bifurcated into two fiber bundles (OC and EC) corresponding to odd (red) and even channels (blue) on the probe face. The 49 detection fibers are ordered in a linear array and imaged onto the CCD and spectrograph (collection bundle)(CC). c) CCD images of i), all 49 channels in ascending order from top to bottom, ii), reflectance image with all odd channels illuminated, and iii), all even channels illuminated. The x and y axes correspond to wavelength and detector fiber position, respectively. d) The LED source with a schematic of the illumination strategy of the 4-LED module, e), the output spectrum of the LED source, and f), a digital image of the raster-scanning imaging platform.

The QDRI clinical system shown in Fig 1A and 1B was developed through collaboration between Duke University and Zenalux Biomedical, Inc. The clinical instrument consists of a desktop computer (Dell, Inc., Plano, TX), a dual-channel high-power LED source (developed in-house), a thermo-electrically cooled CCD and spectrograph (Andor iDUS, Oxford Instruments, PLC, Abingdon, UK), a custom raster-scanning imaging platform (developed in-house), and a custom 49-channel multiplexed fiber-optic probe (Romack, Inc., Irving, TX). The acquisition and control software consists of four integrated modules executed in parallel on a multi-core processor, each developed in LabVIEW (National Instruments, Austin, TX). The imaging probe shown in Fig 1(B) consists of a 7x7 array of fiber-optic channels spaced 6mm apart, each comprised of 8 illumination fibers surrounding a single detection fiber (NA = 0.22, d = 200μm). The effective imaging area is 17cm^2^. The center to center source-detector separation distance for each channel is 700μm. The illumination fibers for each of the 49 channels are collected and subsequently bifurcated into even and odd illumination bundles; the odd and even channels of the probe are illuminated independently. Two self-calibration channels (SC1: odd calibration, SC2: even calibration) are implemented to offset within measurement, intensity variations. The 51 detection channels (49 sample + 2 self-calibration) are arranged in a linear array and coupled to and imaging CCD and spectrograph. A detailed diagram of the probe design is shown in Fig 1(B). A sequential illumination pattern of the odd and even channels is used to reduce inter-pixel crosstalk; two consecutive spectral snapshots are required to obtain a full 49-channel frame as shown in Fig 1(C).

A thermo-electrically cooled imaging spectrograph with a back-illuminated 512×512 pixel CCD camera was utilized for multi-track spectra acquisition. The 49 detection channels of the probe are routed to a single detection bundle wherein all 49 fibers are arranged in a linear array and coupled to the entrance slit of the spectrograph. Each channel corresponds to approximately 8 vertical pixels on the CCD that are binned in software. Pixels corresponding to odd and even detection fibers are binned in two sequential frames, respective to the illumination odd and even sequence, thus reducing the crosstalk between adjacent channels on the CCD. The CCD crosstalk between the adjacent tracks was measured experimentally by lighting up only every other four detection fibers and meauring the counts in the center channel (inactive) between two adjacent active fibers. Fig 1(C) correspond to three images acquired with the 49-ch QDRI system under different illumination conditions. The first panel (i) represents each of the 49 detection channels (in ascending order from top to bottom) illuminated with fluorescent room light and imaged onto CCD showing that all 49 detection fibers are imaged onto the 12.7x12.7mm CCD with minimum “smile” distortion observed on the CCD. The dark area between two tracks is due to the cladding of the fibers. The double peaks of the fluorescence lamp spectrum at 542.4nm and 546.5nm can be resolved, indicating that the spectral resolution is around 3–4nm. The second and third panels (ii and iii) show the spectroscopic images acquired from a flat-white reflectance target with all odd channels on (25 tissue channels plus a SC channel SC-1 at the center) or all even channels on (24 tissue channels plus a SC channel SC-2 at the center), respectively.

A dual-channel LED source was developed to reduce total acquisition time and to improve the light source efficiency. The light source design is shown and diagrammed in Fig 1(D). The source consists of two identical LED modules; each module contains two cool-white high-power LEDs, a cyan LED (λ_peak_ = 510nm), and a blue LED (λ_peak_ = 470nm)(Luxeon Rebel, Phillips Lumileds, San Jose, CA). These four discrete LEDs were chosen to approximate spectrally flat white light from 420nm to 600nm; similar sources do not exist commercially. The LED modules improve the SNR of the system by obviating the use of a thermal source in which the intensity can shift beyond 7%. Each of the 4 discrete LEDs in a single module is coupled to a single illumination bundle on the probe with a polycarbonate concentrating lens (Polymer Optics, LTD, UK). The first segment of each illumination bundle consists of a liquid light guide (d = 5mm) (Thorlabs, Inc., Newton, NJ) which serves to create a uniform spatial distribution from each of the 4 LEDs. The spectral output of the source is shown in Fig 1(E). A microcontroller based mechanical relay control unit is used to interface the computer and selectively power each LED module. The reproducibility of light intensity for this source was determined by acquiring 25 sequential measures using a 99% reflectance target at 10 separate integration times corresponding to dynamic range of the CCD (2–58k counts). The maximum variation observed was less than 2%.

A custom, imaging platform was developed in-house and consists of a pressure sensitive base utilizing 4 discrete force sensors, each with a dynamic range of 1–44 N, a mechanism to physically hold the probe, a digital webcam for specimen-optical parameter co-registration, and a custom computer controlled XYZ raster scanning mechanism. The imaging platform is shown in Fig 1(F). The raster-scanning imaging platform was developed to improve the sampling resolution of the 49-channel device in a controlled manner. This is achieved by incrementally translating the probe in both lateral directions using precision stepping motors (NEMA 17) and subsequently translating the probe downward to establish contact with the specimen placed atop a base with pressure sensors that are continuously polled by the acquisition software. The scanning mechanism can reproducibly (within 1%) move the probe in each direction in increments of 10μm or greater at a rate of 20mm/s; providing an avenue to raster-scan samples on intra-operative time scales.

#### Software Description

**Fig 2:**
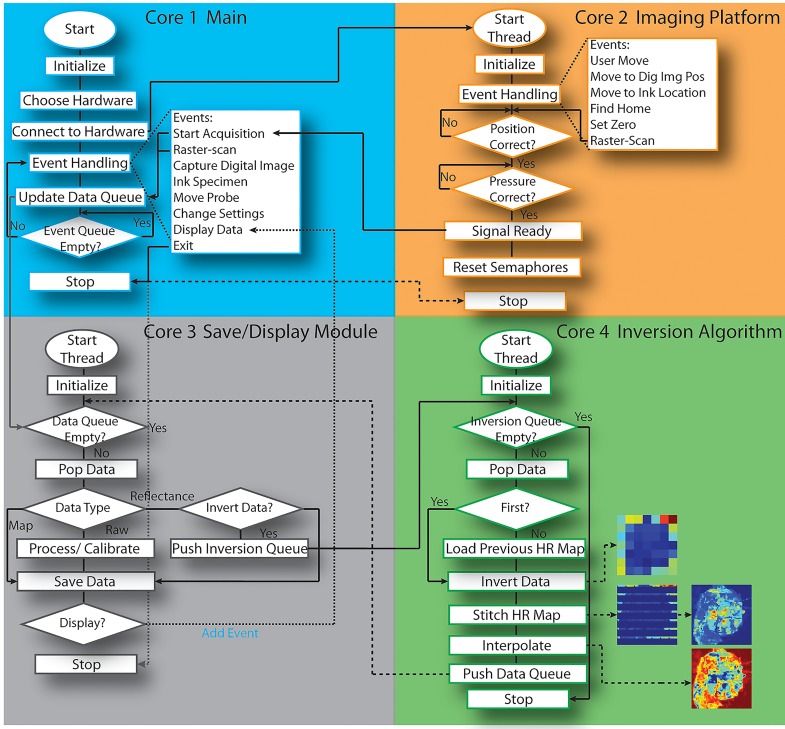
Software Algorithm. A diagram of four discrete software modules, each dedicated to a single core of a quad-core processor (Main, Imaging Platform, Save/Display, Inversions), comprising the spectral imaging software package. Each of the four modules is executed in parallel; inter-module synchronization and data exchange is achieved via semaphores and four dedicated dynamic data queues, respectively.

Four software modules independently executed on a quad-core processor are each dedicated to the four most time consuming asynchronous processes: a main program for user/module interfacing, a platform control module, a data saving and display module, and a spectral fitting inversion module. The main program consists of queue-based finite state machine to preserve system resources and to dynamically determine the next desired action. Also utilized within the main program is a dedicated iteration manager that auto-generates the sequence of actions based on the type of measurement that is desired. For example, if the user wanted to take ten repeated measures across several different integration times as well as light intensity outputs, this is easily achieved by selecting these iterators, in order, from a menu in software at runtime. Furthermore, all tasks are class-based (object-oriented) such that device/system specific actions are dynamically inherited from the appropriate subclass at runtime, meaning that system components, output sequences, saving methods, and inversion routines, can all be modified at runtime. The software was designed in this manner to achieve maximum scalability; the software package is platform and acquisition sequence independent. The imaging platform control module is dedicated solely to controlling the movement output for raster-scanning and pressure control/sensing; independent parallel execution is necessary for this module considering the pressure applied must be constantly polled and compensated. The data and display management module consists of two queues; one queue is dedicated to capturing raw data as it becomes available, the raw data is processed and a second queue is utilized for display purposes. The data is simultaneously saved and pulled from the second queue to the front panel display: each of these processes are non-deterministic and require parallel execution independent from other time-critical processes. The final module is dedicated to intraoperative spectral data inversions (conversion of raw reflectance to optical parameter maps) and is yet another queue-based module. The inverse model utilizes a scalable Monte-Carlo package developed by Palmer *et al* (US 7,570,988) [18, 43, 44, 50]. The inversion routine is primarily MATLAB-based and is called from within LabVIEW. The routine requires 50–300ms per spectrum and is also non-deterministic due to the fitting algorithm; margin-wide optical parameter maps can be obtained within seconds of raw data acquisition and can therefore be used for intra-operative settings. Fig 2 diagrams each of the 4 discrete modules in the context of a single margin raster-scan acquisition.

### System Characterization

#### Signal to Noise Ratio (SNR) and Crosstalk Measurements for Spectral Imaging of Tissues

The crosstalk between neighboring probe pixels (at the distal end in contact with tissue) due to scattering was simulated using a forward Monte-Carlo model developed by Wang *et al* [51]. The maximum observed crosstalk is expected for pixels positioned near the center; these experience contributions from the largest number of neighboring pixels. Simulated results were based on an adipose tissue sample (<μ_s_’> = 6.9 cm^-1^, <μ_a_> = 4.6 cm^-1^, (λ = 450–600nm)) previously determined to involve the highest clinically observed crosstalk by Bydlon *et al* [52].

The SNR was determined using measurements taken on a 127x127 mm, 10% reflective Spectralon imaging target (Labsphere, Inc., North Sutton, NH) 25 times in sequence (n = 25). SNR reported in this work is calculated as 20*log(I_avg_/σ^2^_I_), where I corresponds to the background corrected intensity. Maximum, minimum, and typical SNR values were obtained by modulating the integration time such that the measured average intensity across channels was approximately the highest value within the linear range of the CCD (~58k counts), the lowest value (~2k counts), and a value typically observed during a clinical measurement (~30k counts), respectively.

#### Optical Property Extraction Accuracy

A set of 12 liquid tissue-simulating phantoms with known optical properties was utilized to characterize the optical property extraction accuracy achieved with the 49-channel QDRI system. The tissue phantoms used in the “phantom study” were designed according the methods and protocol described by Bender *et al* [39] and Palmer *et al* [18, 43]. Tissue scattering and absorption were simulated using 1μm polystyrene microspheres (Polysciences, Inc.) and isolated ferrous hemoglobin (Hb) (Sigma-Aldrich, Inc.), respectively. Concentrated hemoglobin was added in ten increasing-volume aliquots to a base non-absorbing phantom with an average reduced scattering coefficient (<μ_s_’>) = 9.73 cm^-1^ (λ = 450–600nm), resulting in average absorption coefficient (<μ_a_>) ranging 0 to 8.86 cm^-1^. The complete summary of phantom optical properties is provided in Table 1.

**Table 1:** Tissue Phantom Optical Properties.

Phantom #	Expected <μ_s_’> cm^-1^	Expected <μ_a_> cm^-1^	[Hb] μM
1	9.73	0	0
2	9.41	1.07	13.89
3	9.12	2.07	26.87
4	8.84	3.01	39.07
5	8.58	3.90	50.62
6	8.34	4.70	61.00
7	8.10	5.50	71.39
8	7.89	6.25	81.12
9	7.68	6.96	90.34
10	7.48	7.63	99.04
11	7.29	8.26	107.22
12	7.03	8.86	115.01

Note: Values are averaged over corresponding wavelengths 450nm to 600nm.

The mass of each constituent was measured and recorded at each step of the phantom fabrication process to reduce random and systematic error associated with pipetting and changes in density caused by temperature fluctuations. Each phantom was thoroughly mixed just prior to being measured to reduce the effects of solution settling. Ten repeated diffuse reflectance spectra were collected for all channels for each phantom. The inverse Monte-Carlo model previously developed by our group was used to invert diffuse reflectance spectra to constituent optical properties (μ_a_ and μ_s_’) [18, 39, 43]. Briefly, the Monte-Carlo model utilizes a non-linear least squares fitting routine to best match a corrected reflectance spectrum to a Monte-Carlo generated reflectance look-up table generated for the source-collection geometry and optical characteristics. Reflectance spectra are scaled by calibrating to a reference phantom with known optical properties; this, alongside a Spectralon calibration measurement to account for the wavelength dependent source intensity, account for the system-specific response. A leave-one-out cross validation analysis was performed using each of the 12 tissue simulating phantoms as a reference phantom against the remaining 11. Extracted values for μ_a_ and μ_s_’ were analyzed as percent error relative to the expected values for each phantom/reference phantom combination.

#### Pressure Optimization

**Fig 3:**
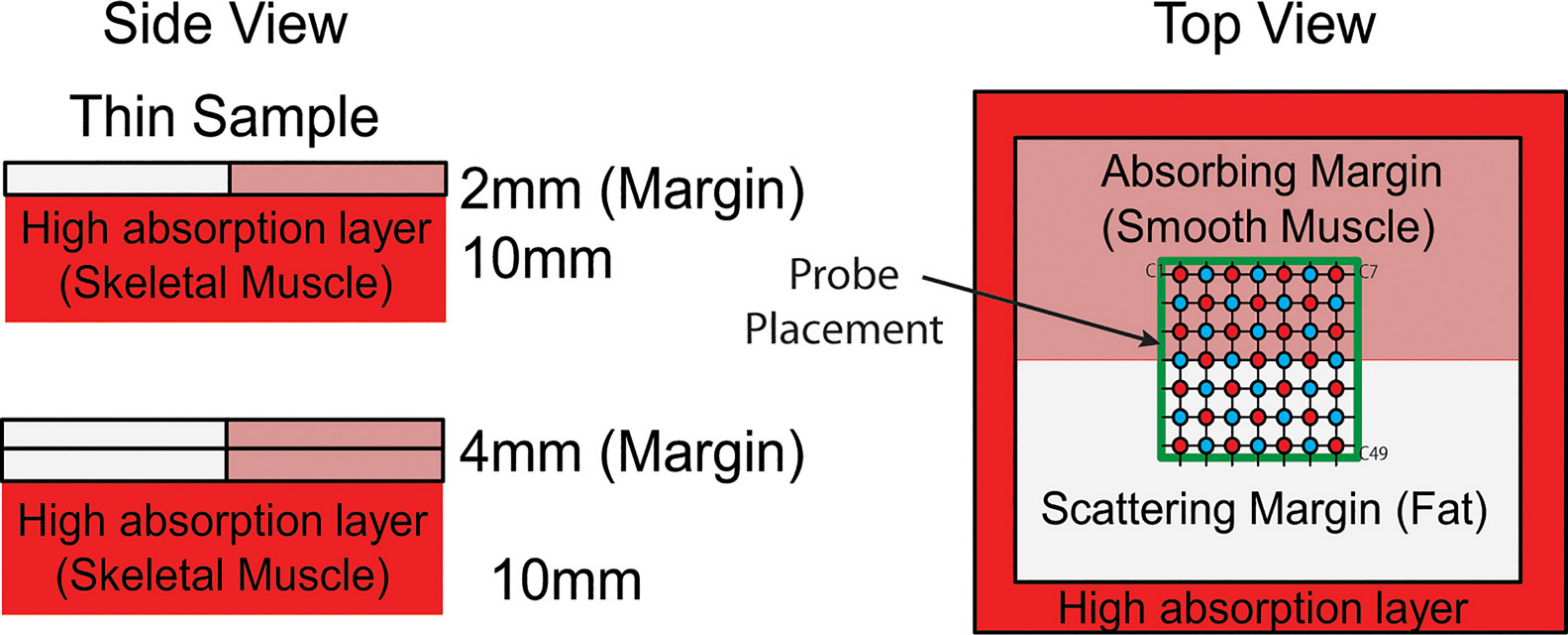
Pressure-testing Assay. Two margin thicknesses are simulated using meat samples with a well-defined thickness. A high-absorption thick layer rests beneath a half-scattering half-absorbing layer and serves as a source high contrast to reveal compression of the top layer.

A layered tissue model was used to examine the effects of pressure at probe-tissue interface as well as inform an optically valid range of pressures for clinical use. A clinical specimen was simulated using 2mm thick slices of meat (bacon) with well-defined adipose (scattering) and muscle (absorbing) regions as “margin” layers, as well as a highly absorbing slab of meat (beef steak) as a “malignancy” at a specific distance beneath the “margin.” The experimental model is diagrammed in Fig 3. The probe was oriented such that half of the pixels correspond to the absorbing half of the top layer, while the other half was used to survey the scattering region. Diffuse reflectance was recorded for 3 repeated measures over a range of applied pressures in 2mmHg increments up to 16mmHg. These spectra were then inverted to obtain the constituent optical properties (μ_s_’ and μ_a_) for each measured region. We repeated this experiment for two margin thicknesses, 2mm and 4mm, achieved by stacking multiple top layer samples.

#### Resolution Characterization

The resolution achieved with raster-scanning was established using a printed 1951 USAF resolution target placed atop a Spectralon target and a pliable scattering medium. The Spectralon target was used to simulate a best-case diffusive background while the pliable medium was used to determine the influence of motion artifacts that may occur as pressure is applied. The pliable medium was tested in two embodiments: white Play-Doh and TiO_2_ infused PDMS. No discernible difference was revealed for measurements of the pliable media relative to each other or to the Spectralon target. The target was then measured as a function of upsample factor, hereafter referred to as “n”; which is a measure corresponding to the number of evenly spaced locations between neighboring pixels. For example, an n = 6 would correspond to 36 (n^2^) placements of the entire probe in increments of 1mm (6 measurements between 6mm spaced channels) in both the x and y directions. Each of the two samples were measured at n = 10, 8, 7, 6, 5, 4, 3, 2, 1.

### Clinical Investigations

#### Clinical Protocol and Patient Population

Diffuse reflectance spectra were collected from excised breast tissue specimens from a total of 32 margins in 20 patients. For the purposes of this work, patients undergoing BCS as well as breast reduction surgery were recruited. Specimen orientation for lumpectomies (partial mastectomies) was determined according to surgically placed reference features including: a surgical wire inserted into the center of the tumor, colored sutures, and surgical clips. Specimen faces were defined as the faces of a cube and labeled relative to the specimen orientation *in situ*; the six measureable faces are hereafter referred to as the superior, inferior, posterior, anterior, medial, or lateral margin. Reduction mammoplasties do not have such a reference system; although several portions of the removed tissue are sectioned and sent to post-operative pathology for assessment, the margin status is irrelevant in these specimens.

Immediately following tissue resection, partial mastectomy specimens were sent to radiology for an intra-operative mammography to verify removal of the tumor mass. Upon return, the specimen was placed onto the pressure-sensing base of the imaging platform and oriented accordingly. It should be noted that in the majority of cases the samples had a pancake-like shape and thus the largest opposing margins were imaged with the C49 system. The pancake-like shape of the specimen is a prominent phenomenon for lumpectomy specimens; the nature of the surgical procedure followed by specimen mammography often results in this shape[53]. Interestingly, this is advantageous in the context of an optical margin assessment tool as roughly 50–85% of the specimen surface area comprises the measurable two opposing margins. Following orientation, the raster-scanning procedure was initiated and the diffuse reflectance spectra were collected across the visible spectrum (λ = 420–700nm). The pressure applied to the face of the specimen is dynamically controlled by a feedback loop that executes in parallel to the main acquisition software, such that subtle adjustments to the applied pressure can be made without interrupting spectrum collection. The specimen was then flipped to its opposing margin and the scan was initiated a second time. The measurement order was determined *ad hoc* because it is not possible to measure a margin multiple times due to time restrictions, nor is it possible to know with certainty the margin that has the highest likelihood of positivity *a priori*. Once the scanning procedure was completed, a “site-level” inking procedure was performed wherein 6–10 sites were randomly marked using tattoo ink (typically orange in color) with the aid of a co-registration structure that physically relayed the central location of each optical channel to the respective point of contact with the specimen. The co-registration plate was then removed and the four corners of the margin were marked with ink of a different color (typically green). A single margin was inked for post-operative pathological assessment in a uniformly spaced diamond pattern. Margin inking was followed by the acquisition of a digital image using an on-board digital camera mounted to the imaging platform. Tissue optical property maps were reconstructed post-measurement using the inverse Monte-Carlo model discussed previously. A board certified pathologist (JG) used these inked dots to provide site-level (orange dots) and gross margin level (green dots) histopathology to which the spectral endpoints at the margin level and site level are compared.

The following characteristics were recorded for each patient (if available): radiographic breast density, menopausal status, age, body mass index (BMI), (chemotherapy or endocrine therapy), prior surgeries and surgical re-excision status. For mammographic breast density (MBD), each patient was assigned a value based on their pre-surgery mammogram: 1 (fatty), 2 (scattered fibrous), 3 (heterogeneously dense), or 4 (extremely dense). For the analyses in this paper an MBD score of 1 or 2 was considered to be low density, while a score of 3 or 4 was considered to be high density; the data was binned in this manner due to the fact that a majority of the patients had scores of 2 or 3.

#### Performance Characterization

Clinical inter-scan reproducibility of the pressure/scanning system was determined through repeated scans of benign reduction mammoplasty specimens. A series of three replicate raster-scans were acquired for three separate reduction mammoplasty specimens intra-operatively at an upsample rate of 8 on a single margin (typically posterior). Between scans, the probe was returned to the start position and cleaned with a disinfectant to reduce the effects of tissue buildup on the probe. The relative standard deviation (RSD) was determined as a percentage by dividing the standard deviation of the three images by the mean of the images and taking the absolute value (RSD = |σ/μ|).

#### Clinical Data Analysis

An empirical cumulative distribution function (eCDF)-based analysis was performed on the 20-patient dataset at the full-frame margin level and at the site-level, where parameter values solely correspond to regions marked for histological validation. Margin level optical parameter maps were segmented using MATLAB to remove regions beyond the boundary of the margin face and was achieved using a simple edge-detection kernel. The eCDF was computed in MATLAB by vectorising the pixels corresponding to either a site-level region or a segmented margin parameter map; these vectors served as inputs to the built-in *ecdf* function. The resulting distribution represents a measure of the optical landscape; the optical landscape serves as a surrogate to the morphological landscape of the tissue data in question. The ratio of β-carotene concentration to the magnitude of tissue scattering ([β-carotene]/<μ_s_’>) was used to characterize the margin landscape of benign and malignant tissue types. To account for inter-patient variations in baseline scattering, the patient data was grouped by mammographic breast density as explained previously. A two-sided Kolmogorov-Smirnov (KS) statistic p-value was used to determine if tissue-specific optical parameter distributions were from a common parent distribution. Benign tissue types were tested against one another while malignant tissue types where tested against individual benign tissue types as well as an average benign distribution. The p-values for each test permutation were used to assess the efficacy of this technique to categorically discern tissue subtypes based on the morphological landscape.

## Results

### System Characterization

#### SNR and Crosstalk

**Fig 4:**
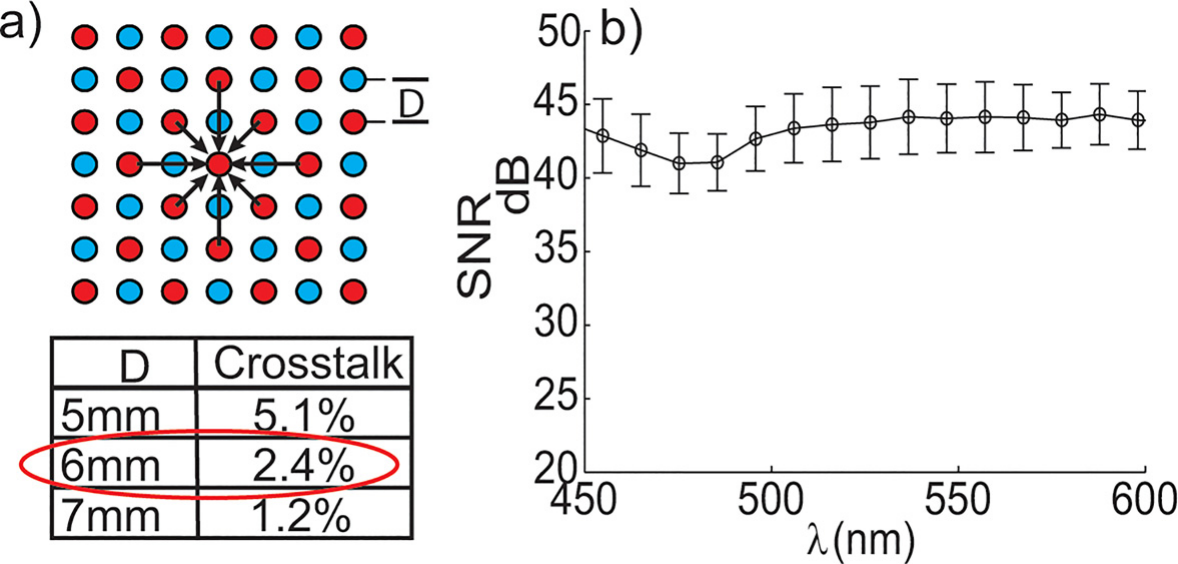
SNR and Crosstalk Characterization. a) Simulated tissue crosstalk comparison for three channel separation distances (5,6, and 7mm), the probe native resolution (6mm) is circled in red. b) The signal to noise ratio (SNR) as a function of wavelength averaged over all 49 channels. The SNR was computed using the equation 20*log(I_avg_/σ^2^_I_) on a 10% uniform reflectance standard.

The optical crosstalk of the system was found to be insignificant for both inter-channel crosstalk and CCD crosstalk. The CCD crosstalk between adjacent tracks was measured experimentally and determined to be less than 1%. This determination was made by illuminating every 4^th^ detection fiber and measuring the counts in the central, inactive fiber. At the native probe resolution (6mm), the crosstalk observed for center pixels was shown to be 2.4% or less and is observed in Fig 4(A). The crosstalk percentage is based on the percentage of signal that did not originate from the pixel in question. Simulated results were based on an adipose tissue sample previously determined to involve the highest clinically observed crosstalk by Bydlon *et al* [52]. Fig 4(B) represents the average and standard deviation of the signal to noise ratio (SNR) as a function of wavelength across each of the 49-channels. The SNR was found to be greater than 40dB across all wavelengths when computed on 10% reflectance standard measured at the integration time corresponding to the measured intensity typical of that seen on clinical samples (~30k counts).

#### Optical Property Extraction Accuracy

**Fig 5:**
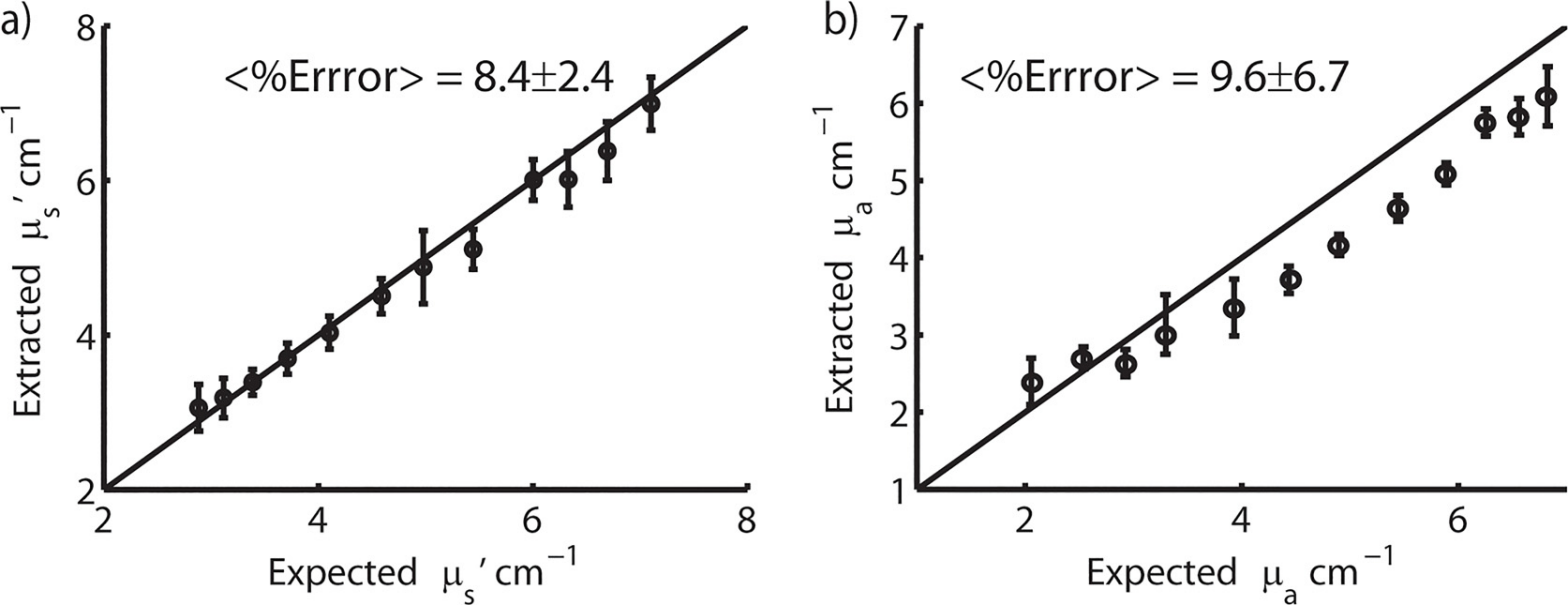
Expected vs. Extracted Optical Properties. Scatter plots of the expected versus the extracted values of μ_a_ (cm^-1^) (a) and μ_s_’ (cm^-1^) (b) for the phantom study set. The diagonal line indicates perfect agreement.

The optical property extraction accuracy was shown to be well within typical error percentages obtained using previous generations of the device (<10%). Using multiple reference phantoms, values for <μ_s_’ (λ)> and <μ_a_ (λ)> were determined to be 8.4 ± 2.4% and 9.6 ± 6.7% of the expected value for the entire span of phantoms measured. Fig 5 illustrates the optical property extraction accuracy with the extracted values plotted as a function of the expected values.

#### Effects of Pressure and Resolution

**Fig 6:**
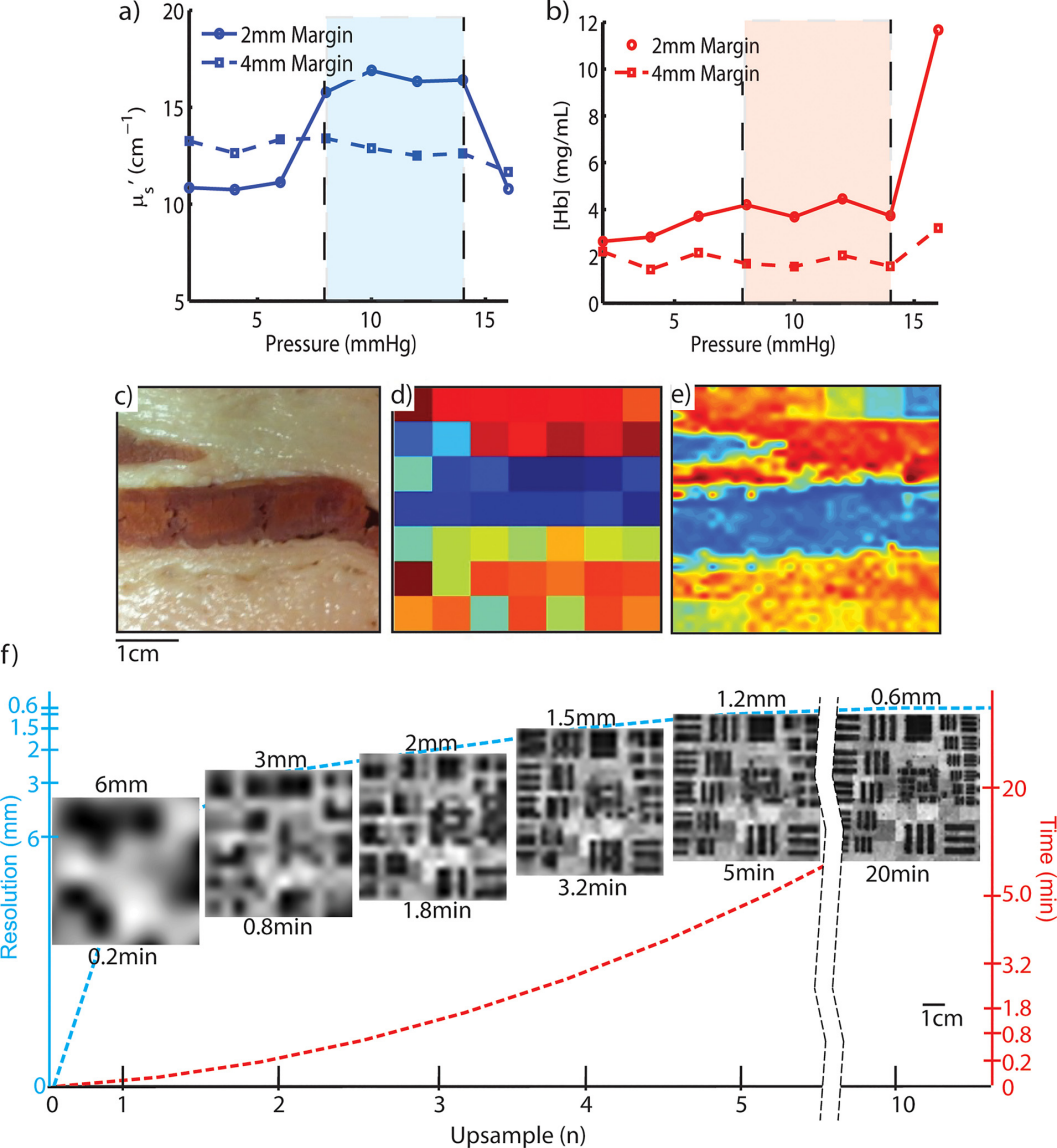
Optimization of Pressure and Resolution. The benefit of controlling pressure shown as a), changes in the reduced scattering coefficient (μ_s_’) as a function of pressure for pixels in the fatty region of the layered meat sample (described in the methods) and b), the corresponding changes in the absorption coefficient (μ_a_) for pixels corresponding to the smooth muscle region. Improvements afforded by increasing the resolution are shown by comparing c), a digital image of a meat sample to d), a hyperspectral reflectance image acquired using the native probe resolution (6mm) and finally to e), a measurement (n = 8), corresponding to a resolution of 0.75mm from 64 (raster-scanned) native probe images. f) Images from an USAF resolution target showing the tradeoff between resolution and time as a function of upsample factor, n.

The pressure applied at the probe tissue interface can substantially alter the observed optical property values. These effects of pressure are well demonstrated using the described meat model to simulate a margin-like sample. Fig 6A and 6B indicates that for the scattering region of the 2mm margin, there exists a narrow pressure window in which the extracted optical parameters are valid. The change between 5 and 10mmHg (Fig 6(A)) represents the point at which contact was established while the change that occurs beyond 15mmHg represents the point at which the margin was compressed; the <μ_s_’ (λ)> value decreases due to absorption that occurs beneath the scattering margin. These changes are not as pronounced in the absorbing region of the top layer (Fig 6(B)) due to the inherent absorption of the top layer; however, a marked increase in measured [Hb] was observed beyond 15mm Hg, suggesting that the highly absorbing bottom layer can still be detected in the presence of absorption in the top layer. The effects of compression are not obvious in the thicker (4mm) top layer specimen within the range of pressures investigated due to insufficient compression.

The ability to discern small regions of contrasting optical properties is substantially improved with increased sub-pixel sampling. The clear benefit of sub-pixel sampling (n = 8) was demonstrated using a meat sample and is shown in Fig 6(C), 6(D) and 6(E). At the native probe resolution, only the regions of highest contrast, specifically the regions of muscle against regions of fat, were distinguished. Conversely, the regions of low optical contrast and small feature size were clearly observed in the raster-scanned optical parameter map of the same sample. The optimum upsample factor (n) was determined by examining the tradeoff between resolution and total acquisition time, shown in Fig 6(F); we chose the highest resolution with which two scans can be obtained (1 for each opposing margin) and the inking procedure can be completed within a reasonable intra-operative time frame (~25–35 mins), as informed by our clinical collaborators. At n = 8, a full scan over the entire probe area (17cm^2^, typically lightly larger than a single margin face) can be acquired in 13.8 mins (1.23cm^2^/min) at a resolution of 0.75mm.

### Clinical

#### Clinical Measurements

Clinical raster-scanned (n = 8) images were acquired for 20 patients, resulting in 64 full-frame spectral images (3136 full-spectrum pixels) for each margin imaged. A breakdown of the patient demographics is shown in Table 2. Average collection time for each margin image was 13.8 minutes; a few additional minutes were needed for the inking procedure. Of the 6 possible margins, 2 were measured (anterior/posterior) in most cases due to the pancake-like shape of most specimens. The ratio of [β-carotene]/<μ_s_’> has previously been established by our group as a reliable diagnostic metric for discerning tumor margins.

**Table 2:** Patient Demographics.

	LBD (MBD = 1, 2)	HBD (MBD = 3, 4)
# of Patients	17	3
Avg. Age (range)	70.6 (60–92)	61 (46–70)
Avg. BMI (range)	30.3 (23.7–47.1)	28.67(18.3–38.1)
Surgical Margin Status		
Negative (>2mm)	14 (44%)	2 (6%)
Close (<2mm)	13 (41%)	3 (9%)
Measured Margin		
Anterior	12 (38%)	2 (6%)
Posterior	14 (44%)	3 (9%)
Superior	0	0
Inferior	1 (3%)	0
Medial	0	0
Lateral	0	0
Avg. Lumpectomy Volume (range) (Ellipsoidal)	23.2cm^3^(7.4–19.4)cm^3^	16.6cm^3^(11.4–33.0)cm^3^

**Fig 7:**
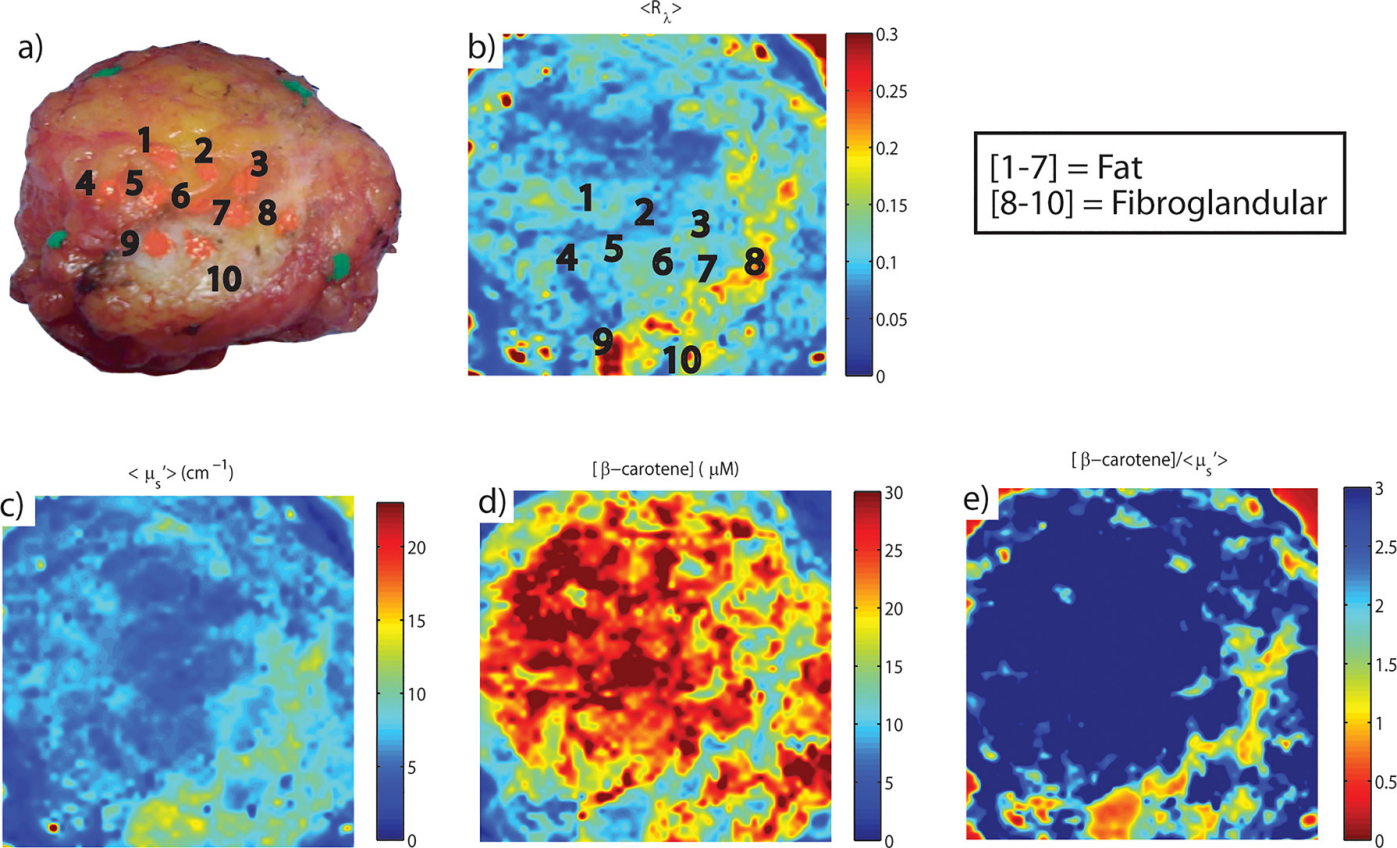
Representative Margin Image for a Breast Conserving Surgery. a) Digital photo showing sites inked for pathological review, b), raster-scanned reflectance map averaged from 450–600nm, c), hi-resolution optical property map of wavelength-averaged μ_s_’, d), corresponding map representing [β-carotene], and e), corresponding ratio map of [β-carotene] to μ_s_’. Sites labeled 1–6 contain mostly fat, sites 7–10 are mostly fibro-glandular tissue.

A representative benign margin is shown in Fig 7. The sites labeled 1–10 were inked and then individually assessed by an expert pathologist (JG). Note the distinct fibroglandular content in the lower right hand portion of the specimen in the digital image of the margin (Fig 7(A)). These types of features (increased fibrous content) are observed as higher reflectance (Fig 7(B)), higher scattering (Fig 7(C)), and lower [β-carotene] (Fig 7(D)). The [β-carotene] to <μ_s_’> ratio emphasizes these changes; there is a multiplicative effect as the tissue transitions from high to low adipose content. Stated another way; the ratio of [β-carotene] to <μ_s_’> decreases as the tissue changes from predominantly adipose to predominantly fibroglandular tissue components. This relationship between optical parameters and benign breast tissue composition has been well established by our group in prior publications [46, 54].

**Fig 8:**
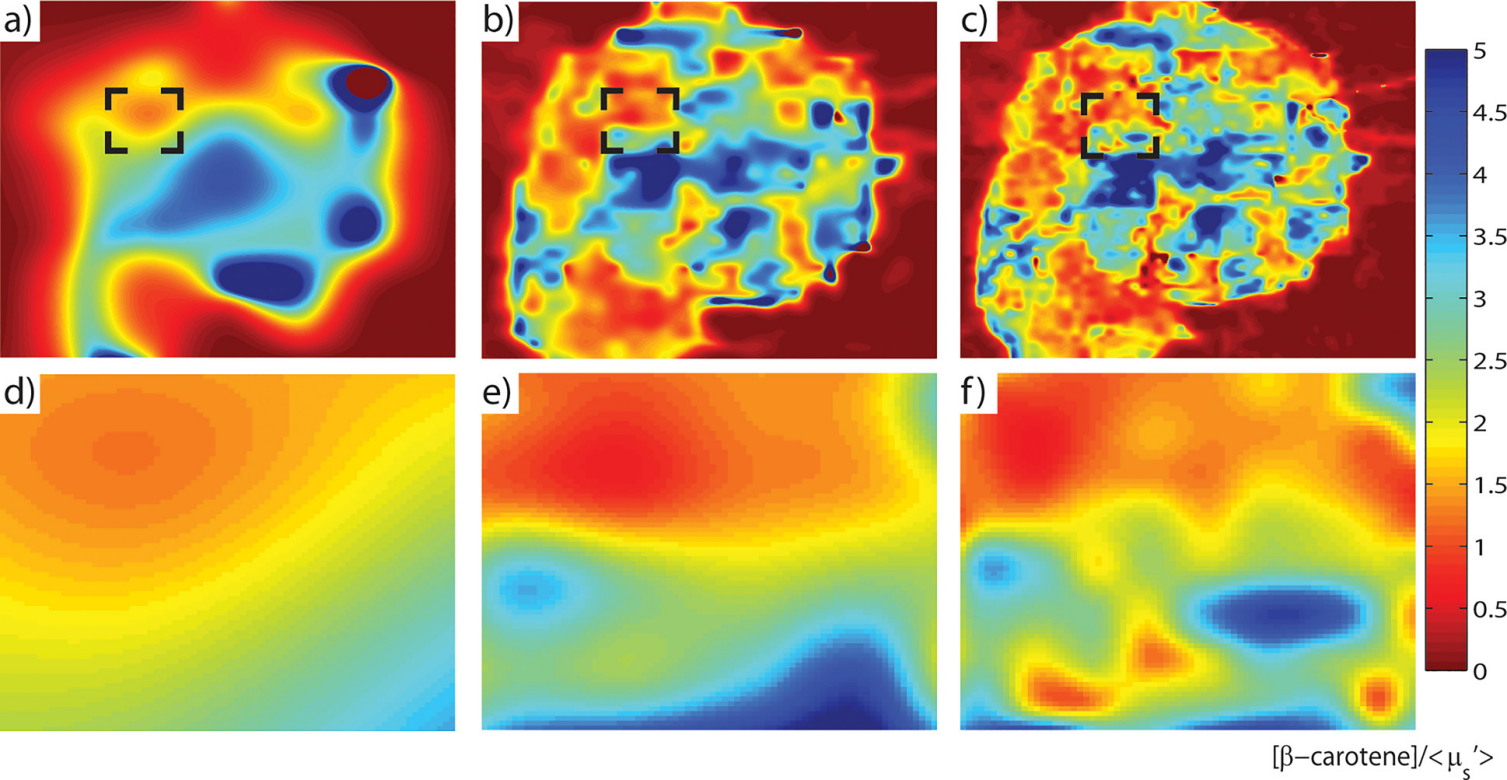
The effect of resolution enhancement of a margin image with a DCIS site. a) Optical parameter map of [β-carotene]/<μ_s_’> at the native probe resolution (6mm), b), at 1.5mm resolution, c), at the best resolution of 0.75mm. d), e), and f), correspond to a zoomed-in version of the delineated site (black box) having confirmed DCIS at the respective resolution.

The enhancement of the probe resolution was shown to have an added benefit in the clinical context: site-level composition accuracy was substantially improved. A margin level [β-carotene]/μ_s_’ map with a corresponding histopathologically reviewed site diagnosed as having ductal carcinoma *in situ* (DCIS) involvement ~1mm from the margin surface is shown in Fig 8. The black box in Fig 8A–8C delineates the area covered by a single pixel and corresponds to a single “site”; there are 49 possible sites for a given margin level measurement. Fig 8D–8F) represent a zoomed version of the marked site in increasing upsample factor. As the figure illustrates, a site having mixed tissue involvement could easily be misclassified at lower resolutions due to the masking effect of the predominant tissue type. At the lowest resolution (6mm) (Fig 8A and 8D), the contribution of the low and high [β-carotene]/μ_s_’ tissue appeared to be evenly split. Conversely, at the highest resolution (0.75mm) (Fig 8C and 8F), several regions of varying [β-carotene]/μ_s_’ were observed.

#### Reproducibility

**Fig 9:**
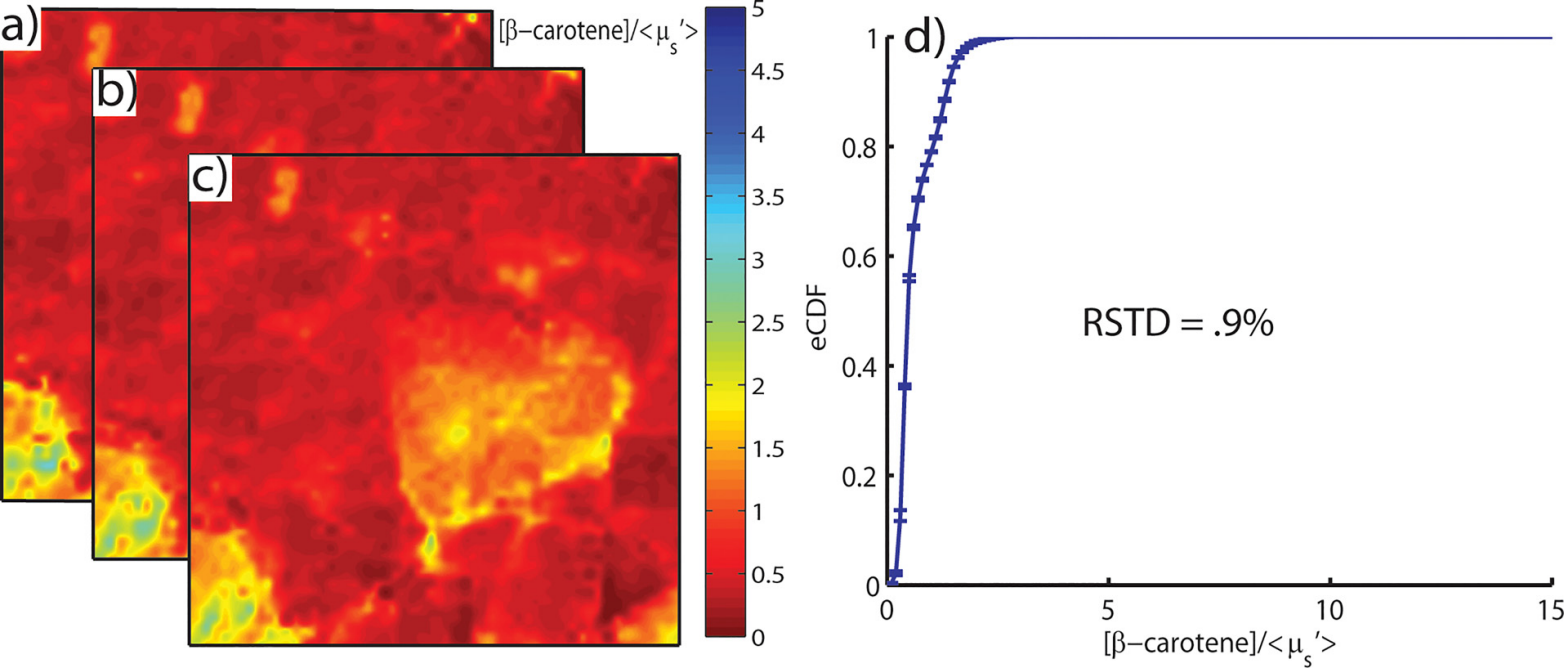
Reproducibility Series. Images a), b), and c) represent 3 sequential raster-scanned [β-carotene]/μ_s_’ maps of a high breast density reduction mammoplasty specimen. Shown in d) is the mean and standard deviation of the cumulative distribution function of each image. The relative standard deviation between scans (RSTD = |σ/μ|) is 0.0092, or 0.9%.

Inter-scan measurement degradation was shown to have a negligible effect on the measured optical properties. Using reduction mammoplasty specimens (9 total margins), raster-scanned parameter maps were shown to be repeatable within 1% for 3 independent scans over 45 minutes. A representative series of independent [β-carotene]/<μ_s_’> maps is shown in Fig 9.

#### Characterization of Benign Tissue Types

**Fig 10:**
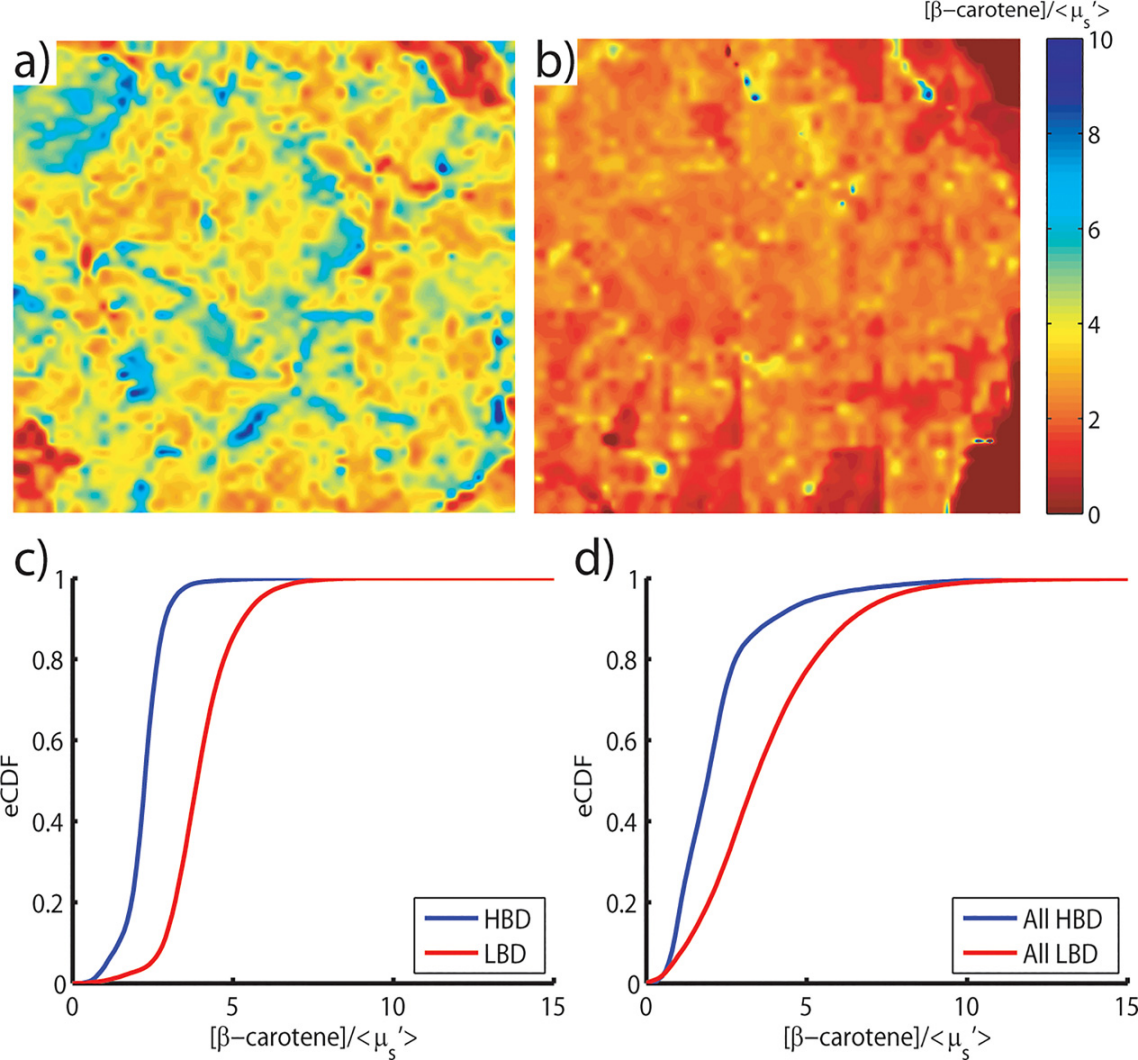
Optical differences in benign breast tissue associated with density. Representative margin level images (maps of [β-carotene]/<μ_s_’>) of a), a low breast density sample (MBD = 2), and b), a high breast density sample (MBD = 3). Cumulative distribution functions stratified by breast density shown for c), the representative margin images, and d), margin level data for all high (MBD = 3, 4)(5 total) and low breast density samples (MBD = 1, 2)(22 total) within the patient cohort.

Relative amounts of adipose and fibroglandular tissue components can be used to quantify and characterize the differences among benign tissue types. These differences are further amplified when considered in the context of breast density. The transition from predominantly adipose tissue to predominantly fibroglandular tissue components manifests as a left shift of the eCDF corresponding to all pixels within a given [β-carotene] /<μ_s_’> parameter map. Representative high breast density (HBD), corresponding to mammographic breast density values (MBD) of 3 or 4, and low breast density (LBD) samples (MBD = 1, 2) and the corresponding eCDFs are shown in Fig 10A–10C.

The lower [β-carotene]/<μ_s_’> values can be explained by the proportions of tissue typical to these breast density grades. Low density breasts are likely to have higher proportions of fatty tissue and therefore present with a naturally higher β-carotene concentration (thereby increasing the ratio). Likewise, high density breasts have higher proportions of collagen and glandular tissue, which manifests as an increase in the scattering signal. These effects are well summarized by the directional shift of the CDFs: fatty tissue associated with the LBD margin has shifted the curve to right; fibrous tissue has shifted the distribution corresponding to the HBD margin to the left. Indeed, these trends were observed to be consistent across the entire patient cohort used in this study, as illustrated in Fig 10(D).

The Kolmogorov-Smirnov (KS) test was used to determine if these distributions are statistically likely to originate from a common underlying distribution. The KS test considers both the shape and size differences when comparing distributions, and is often used in the context of CDFs to quantify the distance between an empirical and a cumulative distribution function and can be considered a goodness of fit. HBD and LBD margins were found to be statistically different (p < 0.02) at the margin level for the cohort used in this analysis.

**Fig 11:**
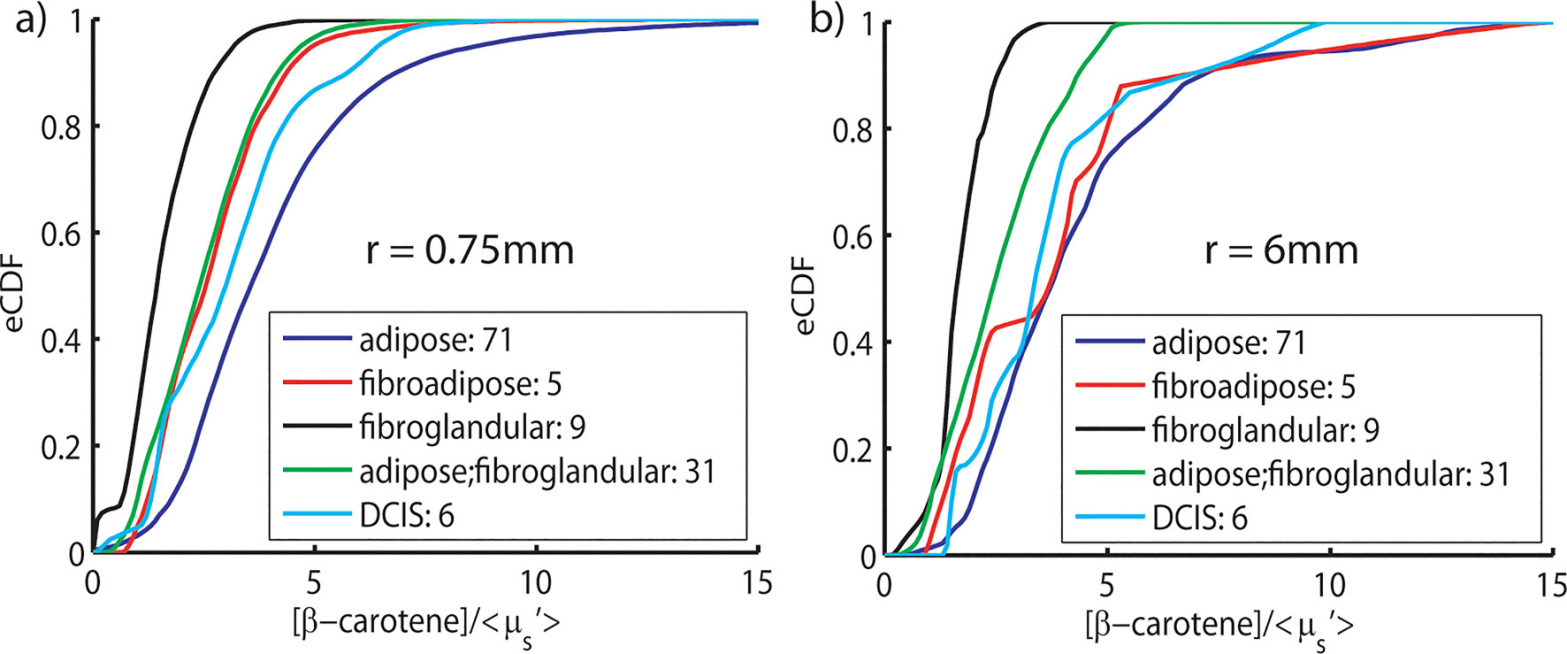
Cumulative probability distributions (CDFs) for all site-level data. Empirical cumulative distributions for eligible pathology confirmed tissue sites. a) Distributions computed from parameter maps with the highest upsample (n = 8) and the best resolution (0.75mm) and, b), distributions corresponding to the native probe resolution (6mm) for the same data set.

A similar analysis was performed at the site level using all available pathology-confirmed sites from the same patient data set. Site designations with n < 5 were excluded from this analysis due to insufficient statistical power. Of the 128 selected sites, 71 were primarily composed of fat (labeled adipose), 5 were a mixture of fibrous tissue and fat tissue (fibroadipose), 9 were a mixture fibrous and glandular tissue (fibroglandular), 31 were a mixture of fat and fibroglandular tissue, and 6 had DCIS involvement within 2mm of the margin. We chose to augment our analysis of site-specific distribution dependencies by also investigating how the eCDFs change when lower/higher resolutions are used. Fig 11 shows the site level CDFs for each of these tissue compositions at the highest and lowest resolution used in this study (0.75mm/6mm).

We observed that as the adipose content is increased, the CDF tends to shift to the right, as suggested by our margin-level analysis. At the highest resolution used, each of these tissue subtypes is statistically distinct from one another. Interestingly, adipose tissues are statistically different from fibroadipose tissues at the highest upsample (p < 0.0001) but not at the lowest, suggesting that the signal from fibrous components is washed out at low resolution. Similarly, fibroglandular mixed tissue and DCIS sites significantly right shift and tend to resemble adipose as the resolution is decreased. The inability to distinguish pure adipose from other tissue types at low sampling resolution suggests that sub-pixel sampling is imperative to accurately determine the underlying tissue composition.

## Discussion

Analysis of our 20-patient clinical data set suggest that the technological advancements leveraged in the C49 system presented here serve to help reconcile the fundamental challenges in achieving rapid yet high resolution, wide field surveillance of breast tumor margins. This is specifically achieved with the development of a wide-field (17cm^2^), low resolution (6mm), 49-channel fiber-optic probe, further comprising a custom raster-scanning imaging platform. The 700μm source detector separation distance for each channel of 49-channel probe reliably affords sensitivity to optical property perturbations from 0–2mm in depth [52], providing sensitivity to malignancy not present at the surface of the excised specimen but within a range correlated with local recurrence. The imaging platform precisely controls the pressure at the probe-tissue interface, reducing the error introduced by the pressure variations characteristic to the user manually applying the probe to the specimen, and affords sub-pixel sampling via incremental translation of the probe in the xyz directions, improving the resolution to 0.75mm on intra-operative time scales. Moreover, the use of a custom imaging platform ensures precise placement of the probe for every measurement and we have shown that the variation from scan to scan is less than 1%. To offset the time required to incrementally acquire full-frame spectral snapshots, a high-throughput dual channel LED source was developed, reducing single frame acquisition by 10 seconds; thus, for a raster-scan with n = 8 (requiring 64 spectral snapshots), 640 seconds are saved, reducing total scan acquisition time to 13.8 minutes from 24.4 minutes, making the device more amenable to intra-operative clinical use. These optimizations are substantiated in the pilot clinical study shown in this work; at the margin-level, tissue morphology is accurately quantified using [β-carotene]/<μ_s_’> as a surrogate for mammographic breast density, which can subsequently be used to stratify and accurately classify site level data. At the site-level, we observed that inadequate sampling resolution can lead to misclassification of tissue subtypes, specifically fibroadipose tissue, a mix of fibrous and adipose tissue, could not be statistically distinguished from pure adipose at the native 6mm probe resolution.

We have shown previously that the technique implemented here is valuable in the context of breast tumor margin assessment due to the high sensitivity to optical contrast of constituent chromophores and scatterers that are direct surrogates of tissue morphology[46]. Interestingly, the study by Brown *et al* revealed that mammographic breast density (MBD) in negative margins exhibited a counterintuitive relationship in that high density breasts corresponded to higher-valued [β-carotene] /<μ_s_’> landscapes, due to a hypothesized increase in baseline [β-carotene] that is characteristic to the smaller adipocytes associated with high density breast tissue. This trend was not observed with dataset presented in this manuscript: although the result seen here is intuitive (since low density breasts are composed of large, non-fibrous tissue, and thus should have increased [β-carotene] and decreased <μ_s_’>, for an overall increase in [β-carotene] /<μ_s_’>), the basis for this discrepancy is unclear at this time. One possible explanation may be related to how the data was grouped: in this study, all tissue was classified as benign, including specimens with a margin status categorized as “close,” having disease involvement less than 2mm from the surface. The decision to group the data this way was based on a recent change in DUMC’s margin classification scheme described earlier, the limited sample size of the pilot study, and the lack of a single truly positive margin accrued within the dataset. Additionally, the patient sample size is limited, even after such grouping, and may contribute to the discordance observed. We speculate the observed trend to be an effect of sampling resolution; however, although some individual specimens revealed a right shift in the eCDF as the resolution was decreased, this effect was not statistically significant for the dataset at large. Alternatively, the result obtained by Brown *et al* could be confounded by variability associated with manually positioning the probe on the specimen surface. Fortunately, outside of the context of breast density, the results between the two studies are in agreement: sites with greater fibroglandular involvement reveal a distinct left shift in the empirical cumulative distribution function.

Kennedy *et al* conducted a study to examine the effects of tissue heterogeneity on the sources of optical contrast described in this manuscript and found no statistically significant differentiation of tissue subtypes (fibroadipose (FA), fibroglandular (FG), adipose (A), carcinoma, DCIS) at the site level using [β-carotene] as a descriptor variable. This shortcoming was attributed to patient demographics, specifically menopausal status [48]. Significant differentiation was achieved for benign tissue types when <μ_s_’> was considered alone, due to fibrous content, again contending that this relationship is likely related to patient demographics. Interestingly, it was found that the total hemoglobin concentration [THb], exhibited the highest diagnostic power. We chose to exclude [THb] from this study due to the post-excisional kinetics of freshly resected tissue specimens as it was determined to be unreliable [55]. Moreover, we found statistically significant differences at the site level for all benign tissue subtypes (FA, FG, A) using [β-carotene] /<μ_s_’> as the descriptor variable. We found that these differences can be obscured at the native probe resolution and therefore attribute the increased sensitivity to the improved resolution obtained with the C49 system.

The importance of resolution is substantiated in a similar emerging technology developed by Lue *et al* utilizing a single channel scanner leveraging diffuse reflectance and laser-induced fluorescence (LIFS) [56]. Although Lue *et al* reported only proof of concept on a single specimen, they were able to demonstrate detection of a region of DCIS > 1mm^2^ using [β-carotene] and collagen scattering as descriptor variables. The point-scanning technology is fundamentally similar to our approach and demonstrates what can be achieved in terms of spatial resolution when the specimen is sampled at very small increments (0.25mm). A raster-scan with n = 24 of the C49 system would yield equivalent spatial resolution to that achieved with the point-scanning system. An important advantage of our technique is the wide coverage area that is achieved in a single acquisition; the simultaneous acquisition of 49 channels over this area theoretically reduces total acquisition time by a ratio of 49:1. Additionally, the C49 system device makes physical contact with the specimen; this is advantageous in the context of the subtle pressure that is needed to flatten the surface of the specimen for imaging. Surface contact also reduces the effects of inconsistent illumination geometry and specular reflection from highly irregular surfaces such as those familiar to most clinical breast specimens.

A multi-separation spatially offset probe based utilizing Raman spectroscopy developed by Keller *et al* has shown promising clinical utility, achieving high sensitivity and specificity (> 94%) on frozen-thawed specimens [57]. Compared to that technology, notable differences of our device include the use of visible-wavelength low-power optical sources, high-SNR source/detection equipment, and the ability to quickly collect and survey the entire tumor margin. The Raman technique relies on a phenomenon that is characteristically difficult to detect and utilizes a point probe, covering an area around 1cm^2^. A technique utilizing OCT by Nguyen *et al* had achieved similar sensitivity and specificity (100% and 82%, respectively). An important distinction regarding this technique is the subjective analysis of information related to light scattering of the tissue at specific depths; this prevents automated quantification of the likelihood of disease [58]. A number of studies have recently been published based on spatial frequency domain imaging (SFDI). SFDI is a wide-field technique that can similarly obtain tissue optical properties by measuring the intensity of diffusely reflected light [59–61]. One presumed advantage of this technique is the non-contact nature of SFDI; however, it has been shown that the surface profile of irregularly shaped breast specimens causes significant error in determining the inherent tissue optical properties. SFDI suffers from low SNR at spatial frequencies corresponding to short photon path lengths, potentially leading to the misclassification of small regions of focal disease.

We have shown that DRS-based techniques are sensitive to underlying tissue morphology and that such sensitivity is crucial in accurately quantifying the underlying margin landscape, and thereby detecting shifts within it. The fundamental limit with such a technique is the loss of sensitivity that occurs when the site in question is not homogenous, particularly in the case of a site composed of less than 25% malignancy: small regions of heterogeneity are washed out with volume averaging. This is particularly important in the context of DCIS, wherein small tentacle like structures can protrude from a centered malignancy all the way up to the margin surface with cross-section on the order of the size of a few cells. Indeed, these types of positive margins often go undetected even during histopathological review as clinical specimens are sectioned at 3–4mm increments; the extent of DCIS involvement can be lost in the sections of tissue not included for pathological assessment. The raster-scanning implementation seeks to address this limitation by oversampling the specimen such that small regions of heterogeneity are highlighted in the presence of homogeneous counterparts. Although a 0.75mm sampling resolution still does not qualify as microscopy, it does provide a highly pragmatic technological intersection addressing the limitations of the native wide-field spectroscopy system and microscopy, and provides a 3-fold improvement in terms of the amount of tissue reviewed during histopathology. Sampling of a full margin currently requires 13.8 minutes, significantly longer than the 30 seconds required for a single, 6mm resolution spectral snapshot, however, this still is within the timing constraints warranted for an intra-operative tool: the amount of time elapsed between specimen removal and surgery completion is typically between 25–35 minutes. The pancake-like shape of most lumpectomy specimens typically warrants measurement of only two margins as the majority of the specimen surface area constitutes the anterior and posterior margins (50–85%), however, the time required to raster-scan a margin will ultimately become a bottleneck if more than 2 are ever to be measured. Furthermore, although we precisely control the pressure applied to the specimen face, we use a standardized 10mmHg pressure as informed by our studies on meat samples, which may not be suitable for all breast tissue subtypes. A future study could include the effects of pressure in the context of mammographic breast density, tissue subtype, and patient BMI.

In conclusion, these studies suggest that quantitative optical spectral imaging may be a pragmatic solution to the margin assessment problem and could ultimately be integrated into the clinical standard of care. We have demonstrated that a multi-channel scanning technique is capable of providing rapid, sub-millimeter resolution surveillance of the tumor margin landscape, which accurately reports on the underlying histopathology. The system presented here demonstrates the clinical translatability of the device, as it was developed in collaboration with a commercial partner, although large scale production will likely require additional optimization. Ideally, the system would consist entirely of low-cost, off the shelf components. A 100-patient prospective validation study is currently underway to robustly demonstrate the diagnostic clinical utility of the technology.
